# From local to global: a qualitative review of the multi-leveled impact of a multi-country health research capacity development partnership on maternal health in Sudan

**DOI:** 10.1186/s12992-016-0153-0

**Published:** 2016-05-16

**Authors:** Khalifa Elmusharaf, Hanan Tahir, Diarmuid O’ Donovan, Ruairi Brugha, Mamoun Homeida, Amal M. O. Abbas, Elaine Byrne

**Affiliations:** University of Medical Sciences and Technology, Khartoum, Sudan; University of Limerick, Limerick, Ireland; National University of Galway, Galway, Ireland; Royal College of Surgeons in Ireland, Dublin, Ireland

**Keywords:** Health research capacity strengthening, Global partnerships, Networks of action, Sudan, Participatory research

## Abstract

**Background:**

There is a substantial body of literature on the principles of good partnerships and the rationale for such partnerships in research capacity strengthening. This paper illustrates the long term effects of a multi-country (8 countries) global partnership for health systems research capacity development (Connecting health Research in Africa and Ireland Consortium - ChRAIC) in relation to its contribution to capacity strengthening, public advocacy and policy influence at different levels and its practical achievements in Sudan in addressing access to maternal health services.

**Methods:**

The authors (all members of the global partnership) reflect on the project in one of its’ partner countries, Sudan, over its’ five year duration. This reflection is supported by specific project data collected over the period of the project (2008–2014). The data collected included: (i) 6 monthly and annual donor reports; (ii) a mid-term internal and end of project independent evaluation of the entire project, and; (ii) a Ph.D study conducted by a member of the Sudanese research team.

**Results:**

The ChRAIC project in Sudan achieved the deliverables set out at the beginning of the project. These included a national knowledge synthesis report on Sudan’s health system; identification of country level health systems research priorities; research capacity assessment and skills training, and; the training and graduation of a Sudanese team member with a Ph.D. Mechanisms established in Sudan to facilitate these achievements included the adoption of culturally sensitive and locally specific research and capacity strengthening methods at district level; the signing of a Memorandum of Understanding at country level between the Ministry of Health, research and academic institutions in Sudan, and; the establishment of country level initiatives and a research unit. The latter being recognized globally through awards and membership in global health forums.

**Conclusion:**

We surmise that the ‘network of action’ approach adopted to partnership formation facilitated the benefits gained, but that adopting such an approach is not sufficient. More local and contextual factors influenced the extent of the benefits and the sustainability of the network.

## Background

Within the last two decades “there has been a burgeoning of global organisations, partnerships, initiatives, and meetings – all focussed on strengthening aspects of health research for development across the globe, and each proposing a different route to this end” [1]. There have been at least three conferences since 2000 to facilitate dialogue and debate on this topic – Bangkok, 2000 (International Conference on Health Research for Development), Mexico Summit 2004 (Ministerial Summit on Health Research), and Bamako 2008 (Global Ministerial Forum on Research for Health). The First Global Symposium on Health Systems Research held in Montreux (2010) called for ‘a new international society for health systems research, knowledge, and innovation’. This symposium was the launch pad for the now highly active Health Systems Global network and has held more conferences since then (Beijing 2012; Cape Town 2014, and; Vancouver 2016).

Additionally, there is a substantial body of literature on the principles of good partnerships and the rationale for such partnerships in research capacity strengthening [2–9]. Guidelines on how to obtain the benefits of partnership can be categorised into three main areas: institutional support, individual support, and improvement of the research environment. Issues of transparency, mutual trust, communication, and dissemination underpin these principles. The main challenges facing partnerships which are debated in the literature are around setting the research agenda, power and ownership, access to and control over funding, capacity imbalances and rewards or benefits of the partnership [10–13]. With the burgeoning of these partnerships there is the need to monitor and evaluate what is happening as there is the danger that partnerships will remain “… a ‘feel good’ panacea for governance without obtaining a pragmatic grasp of the ‘why’ and a clearer understanding of the ‘how’ of partnerships” ([14], p.2).

This paper reports and reflects on the processes whereby national key decision and policy makers and academic staff in one of the African countries, Sudan, that was part of the Connecting health Research in Africa and Ireland Consortium (ChRAIC^1^) embarked on a health systems research capacity development project.

ChRAIC was an African/Irish health systems research capacity strengthening consortium (2008–2015) established with the aim of supporting the Irish Government’s pro-poor development policy through conducting research that would strengthen health systems in Africa [15, 16]. ChRAIC had five main aims: support African Higher Education Institution (HEI) partners to summarise existing research and knowledge gaps on specific components of health systems’ capacity to deliver interventions for the Millennium Development Goals (MDGs) 4, 5 and 6; establish a doctoral training programme; assess and strengthen African HEIs’ research capacity; conduct Irish Aid-relevant research; and strengthen research into policy links.

The partnership comprised three Irish HEIs and counterpart HEIs or research institutions in six African countries: Lesotho, Malawi, Mozambique, Sierra Leone, Sudan and Uganda. After 2012 both the Republic of the Sudan and the Republic of South Sudan were included, expanding the partnership to eight countries. Support to the partnership also came from the Malaria Consortium in Uganda and Sudan and two organisations based in Geneva: the Alliance for Health Policy and Systems Research and the Council on Health Research for Development. This health research capacity collaboration was co-funded by Irish Aid through Ireland’s Higher Education Authority.

The ChRAIC project had two main components. First, a four year Ph.D programme on health systems research co-hosted by the Royal College of Surgeons in Ireland (RCSI), Trinity College Dublin and the National University of Ireland, Galway was developed. Eight students registered for the programme over a period of three years (2008 to 2010), and one of the students was from Sudan. The taught component for the first year of the PhD programme was conducted in Ireland, students returned to the country where the study was to be conducted (usually the home country of the student) for year 2 and 3, and in year 4 the student returned to Ireland to write up and finalise the thesis. The second component of the project was where lead institutions were identified in each partner country and through these institutions research teams were established on a country-by-country basis. The research was divided into two main phases: a) a national-level knowledge synthesis and research capacity analysis to establish research priorities and identify capacity strengthening needs, and; b) based on identified research priorities to plan, allocate funding and implement capacity building and/or research activities.

Specifically, this paper illustrates the achievements of this partnership in Sudan in relation to capacity strengthening, public advocacy and policy influence at different levels and its practical achievements in Sudan in addressing access to maternal health services. However, before detailing these achievements we outline how and why the network was conceived, then discuss the practical outcomes of the project in Sudan, and finally reflect on those outcomes.

## Methods

A reflective practice approach was used to monitor the impact of the ChRAIC partnership on health research capacity development throughout the life of the project. Due to the cyclical and iterative nature of the reflective cycles that took place Gibbs [17] model of reflection (Description, Feelings, Evaluation, Analysis, Conclusion, Action) best describes the process undertaken. Though the reflective process applied to the entire project, this paper examines the reflections specific to Sudan.

These reflective cycles took place in annual cross country meetings, quarterly steering group meetings, and through the six monthly and annual reports to the funder. Additionally, a midterm evaluation of the partnership by the ChRAIC project coordinator and an end of term independent evaluation of the ChRAIC project informed our reflections. At the country (Sudan) level additional data was available from the data collected in South Sudan as part of a Ph.D thesis by the Sudanese student who was a member of the ChRAIC team. This data included: Participatory Ethnographic Evaluation and Research (PEER) – 42 interviews conducted by 14 locally trained women and 42 debriefing sessions conducted with these women; 13 critical incident cases, and; 37 stakeholder interviews [18]. The knowledge synthesis and research capacity assessments in Sudan also assisted with the reflection, especially in the analysis phase.

## Conception of ChRAIC

### What’s in a name?

*Partnership, what a wonderfully elastic concept, with expected strands of equality and shared ownership and threads of equal access to money, power and recognition!* ([19], p. 75)

There are numerous definitions of partnerships and often the term is used synonymously with networks, consortiums, alliances, coalitions, or collaborations. The term has connotations of inter-linkages, exchanges, common goals or processes, forums for discussion, fairness, and the aim to develop the capacity of the individuals and to increase the influence or impact of the research results [10].

ChRAIC was conceived as what could be more appropriately be termed a ‘network of action’ [20]. Networking enables the sharing of experience, knowledge and technology and thereby scaling or generalising from the learning process. The ‘network of action’ principle is based on the “recognition of the need to situate the action within networks rather than on singular units” ([21], p341). Braa et al. [21] argue that the need to develop an institutionalised and sustainable system is not a luxury, but a necessity. Local interventions need to be part of larger interventions. Networks of action are characterised as:

*(i) abandoning singular, one-site (typically one organization) action research projects in favour of a network of sites, (ii) generating local, self-sufficient learning processes together with working mechanisms for the distribution of appropriately formatted experiences across sites in the form of vertical and horizontal flows, (iii) nurturing a robust, heterogeneous collection of actors likely to pursue distinct, yet sufficiently ‘similar’ … agendas, and (iv) aligning interventions with the surrounding configurations of existing institutions, competing projects and effort as well as everyday practices*. ([21], p359)

This understanding of a partnership as a ‘network of action’ goes beyond describing the composition of the group and the projects undertaken. It  includes a multi-layered heterogeneous approach which focuses on the process of conducting and developing the capacity to do the research. The aim is to  achieve more than we can as individuals and to have influence  beyond the pilot site. We return to this concept of ‘network of action’ later on in the discussion.

### Why partner?

There are numerous advantages associated with research partnership approaches involving northern and southern partners. One of the main arguments, in response to past domination of the field by northern researchers, is that it is the ethical and right thing to do. Previous approaches, such as the ‘mosquito’ or ‘parachute’ approaches – names used to describe external researcher(s) or research teams using lower income countries as no more than data collection or testing sites and then leaving with the data to analyse, write up and disseminate elsewhere [22, 23] - were exploitative. Additionally, the sharing of experiences, getting support of outsiders and developing capacity networks, have public advocacy value within countries, regions and globally [10]. Regional research networks, alliances, partnerships, and institutions also have the potential to be powerful entities for lobbying regional development agencies as well as government [11, 24]. The power to influence local policy is greater if there is local ownership of the programme [25]. Networks contribute to regional research findings which have more influence on national and regional agendas when coming from a group of well-known researchers than from a single researcher or institution [11]. Partnerships have the advantage of being able to raise sensitive issues in a more diplomatic manner [24].

Partnerships can develop research capacity through training, mentoring or sharing skills while  conducting a research project [26]. For resource poor countries the financial and human resources and the institutional capacity of the other partners are beneficial, whereas in high-income countries opportunities arise to develop understanding and research into diseases/scenarios not otherwise available to them [12]. Added value comes through the development of networking skills from being involved in the partnership [11]. The pooling of resources and skills is especially important, given difficulties in retention and the resultant scarcity of skilled human resources in most low income countries [11, 24]. Networks can also create a central liaison point for donors, policy and decision makers and other researchers [11]. The inclusion of individual researchers with little institutional support can also decrease feelings of isolation [24].

In summary, there are many benefits described to working in a networked manner. Sometimes this is a requirement for funding or research ethic committees, but other reasons are that it: is the ethical and right thing to do; has public advocacy value; can strengthen research capacity; can pool human, institutional and financial resources, and; can provide support to individuals working in isolation.

## Sudan ChRAIC team

### Sudan

Civil war, political instability, and natural disasters had characterised life in Sudan and hampered economic progress for many years. Additionally, years of conflict consumed much of the country’s resources and had a direct negative effect on the health system, contributing significantly to the country’s low health indicators and slow progress towards the MDGs. There were two main civil wars in Sudan in the twentieth century. The first civil war (1955–1972) was between the northern part of Sudan and the southern region that demanded representation and more regional autonomy. However, the agreement that ended the war in 1972 did not fully dispel the tensions that had originally caused it and the conflict resumed again and lasted from 1983 to 2005. Sometimes the period between 1955 and 2005 is considered to be a single war with an 11-year ceasefire.

In 2002, peace negotiations commenced between the government of Sudan and the Sudan People's Liberation Army in southern Sudan. A preliminary accord in July 2002 provided for a referendum at the end of a six-year period of self-rule in southern Sudan to determine whether or not the region would secede [27]. In 2005, a ‘Comprehensive Peace Agreement’ was signed and granted the southern Sudanese the right to decide if southern Sudan should declare its independence from Sudan [28]. In the referendum in January 2011, 98.83 % of the population voted for independence [29]. On 9 July 2011, the Republic of South Sudan gained independence after five decades of war, conflict and fragility.

### Formation of team

In relation to the research project (2008 to 2015), the political, historical and infrastructural context of Sudan meant that the initial plans for partnering as laid out in the research proposal in Sudan had to change. Both Sudan and South Sudan were experiencing on-going instability, conflict and humanitarian crises, despite the Comprehensive Peace Agreement signed in 2005 and the secession of the South in 2011. Therefore, for the duration of the project flexibility needed to be built into the partnership agreements: from the formation of the local research teams, to meetings and trips being rescheduled and dates for reports or activities being renegotiated. Flexibility was particularly needed for the Ph.D student who was from and lived in Sudan and was conducting research in the border regions of South Sudan.

Initially, the contact institutions for ChRAIC in Sudan were the Malaria Consortium based in Khartoum and the University of Juba, Sudan. The Malaria Consortium closed its Khartoum offices in January 2009, and the university was in the process of relocating from Khartoum to Juba as the situation grew more stable in the south. An opportunity arose to develop a new partnership when a Sudanese candidate was selected to be one of the first intake of ChRAIC Ph.D students. At the invitation of the founder and a senior staff at the University of Medical Sciences and Technology (UMST), a visit to Khartoum was made by two members of the global ChRAIC steering group. During several meetings with UMST, the Ministry of Health (Federal and State), University of Juba, the World Health Organisation (WHO) local office, other potential academic partners and health service providers the way forward was discussed.

As a result of this visit, and at the specific request of the then Sudanese Minister for Health, the recommendation of having two teams (one for North and one for South Sudan) was adopted. UMST agreed to be the lead Northern partner. Existing capacity (human resources and structural) deficiencies at the University of Juba made it difficult for the University of Juba to be the lead of South Sudan ChRAIC partnership. However, after a ChRAIC workshop in 2010 in Kampala, the Ministry of Health of the Government of South Sudan agreed to be the lead partner with the support of the staff at the University of Juba. Additionally, the Malaria Consortium had opened an office in Juba and had the capacity to support the Ministry of Health in financial management and agreed to provide the assistance of one of the Malaria Consortium staff. Thus, three agreements were drawn up by RCSI for the ChRAIC Sudan team: one with the UMST for the North; another with the Ministry of Health, Government of South Sudan for the South; and the third agreement with the Malaria Consortium to support the Government of South Sudan and provide additional support to the overall ChRAIC programme.

The agreement between RCSI and the UMST was signed in October 2009. The main activities laid out in this agreement were in relation to:

- Producing a synthesis of knowledge (published and unpublished) in the areas of: governance of the health system, access and equity to health services, and human resources for health with respect to achieving MDG goals 4, 5 and 6 for Sudan.
- Generation of research priorities for Sudan based on the gaps identified  in the knowledge synthesis.
- Identification of the capacity gaps that needed to be addressed  to conduct the research.
- Addressing these capacity gaps so that the research could be conducted to address the identified research gap.

In July 2010, after a transfer of funds problem was resolved^2^ a series of meetings took place between representatives of UMST and the Federal Ministry of Health (FMoH) Sudan to select the appropriate partners for the knowledge synthesis activity. A Memorandum of Understanding between UMST and the FMoH enabled access to documents needed for the knowledge synthesis. At a later date (August 2010) UMST developed a Memorandum of Understanding with the Epidemiological Laboratory (EPI LAB). In 1997, EPI LAB was established as a public health outreach research centre initiative and as  a non-governmental, non-profit organisation based in Khartoum, Sudan. EPI LAB’s main role in Sudan was to support and evaluate public health programs, strengthen research capacity and link academia to public health. EPI LAB assisted the ChRAIC country team particularly in accessing data on HIV/AIDS and TB.

The uniqueness of signing Memorandum of Understanding between government departments and academic institutions is signified in a comment from one of the Sudanese group members in the midterm evaluation of the ChRAIC programme.

*When we signed the Memorandum of Understanding with the Federal Ministry of Health we invited the Representative of the WHO and in fact in that meeting he said this was the first time in almost twenty-five years a university managed to have a Memorandum of Understanding with the Ministry of Health; and I think that was a very positive point, so the Memorandum of Understanding was very instrumental in engaging all these people in this project. (ChRAIC North Sudan member, Mid-term Evaluation)*

In July 2010 in Khartoum a technical working group was formed and lead by UMST. Other task force and working group members were from the FMoH and the Sudanese Academy for Young Scientists. In all ChRAIC Sudan membership was from:

- UMST
- National Ministry of Health
- The University of Khartoum
- Public Health Institute in the National Ministry of Health
- University of Sciences and Technology
- The National Laboratory of Sudan

The steering committee of ChRAIC North Sudan had a balanced female/male representation and this gender balance was seen across the broader multi-country ChRAIC partnership regarding the composition of the teams, opportunities for participation, and the generation of outputs.

**Fig. 1:**
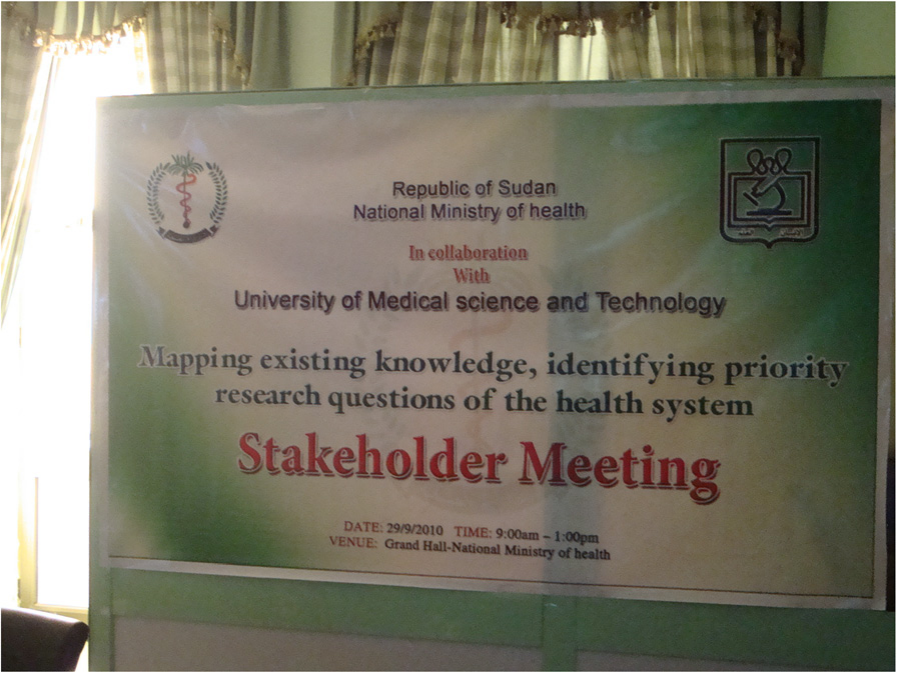
Banner for the Stakeholder meeting in Sudan

A stakeholder’s workshop was planned as the first activity in Sudan as it was considered to be important to bring stakeholders together before commencing the knowledge synthesis and research priority setting (Fig. 1).

**Fig. 2:**
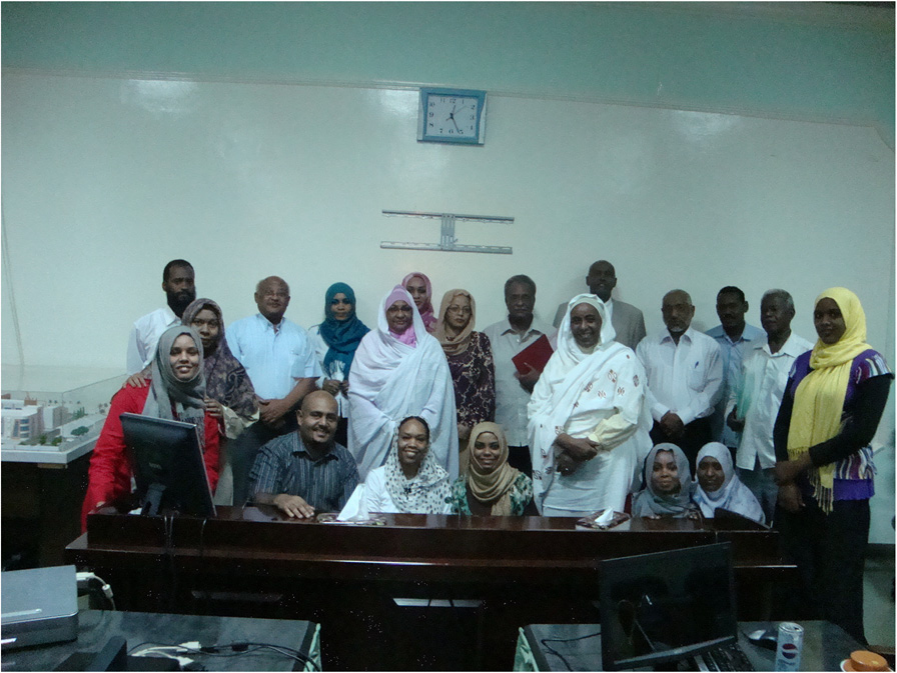
Technical and core working group/stakeholders

The stakeholder’s workshop took place on the 29th of September 2010 in Khartoum, Sudan and invitees included representatives from all Sudanese academic institutions interested in health research policies, Non-Governmental Organisations (NGOs), and the ethical committee at FMoH. A proposal for conducting the knowledge synthesis was prepared by a technical committee and distributed to all participants for discussion. The participants suggested that it would be important to conduct a systematic review of the available national databases and study reports, but also to include key informant interviews. These key informant interviews would be useful in finding out if there was any grey literature missing from the review, but also would be a suitable means of obtaining views of the different stakeholders (Government, community, NGOs) about research priorities (Fig. 2).

## Results

### Activities completed

#### The knowledge synthesis and research publication

A road map for conducting the knowledge synthesis was drawn up in the stakeholder meeting. Subcommittees were formed for the technical and core working groups and each subcommittee was assigned part of the knowledge synthesis exercise: one on governance, one on human resources and one for equity and access. Despite the fact that all members of the technical working group and the core group were extremely busy, they scheduled to meet every Monday to conduct the knowledge synthesis.

The process of data collection and final knowledge synthesis report writing went smoothly for the period December 2010- March 2011. However, the tensions experienced across all parts of Sudan before the referendum to separate the North and South resulted in some instability in the FMoH. Since some of the North Sudan ChRAIC team members were from the FMoH this impacted negatively on the process of report completion. However, the process of completing the knowledge synthesis was thorough and well documented and was in itself an important outcome from the project.

*…. the great thing about this project is not only the deliverables of the project, it’s the process itself. We are using a number of methodologies to engage policy makers, officers, academia, in one place to think about this project and to do this project. So I think the great thing about this project is the process and the methodology of conducting it, I think it’s unique. (ChRAIC North Sudan member, Mid-term Evaluation)*

The knowledge synthesis was completed in January 2013. The entire report was not disseminated in one event but targeted to particular events and activities. For example, the section on the Human Resources for Health was disseminated by the Public Health Institute in the 2nd issue of their newsletter “The Evidence” and referred  to the Sudan ChRAIC knowledge synthesis report as a “key document for human resources for health research situation analysis, priority setting, and strategy development”. The Knowledge synthesis process and the report are also referred to in the Sudan Health Research System Policy and the prioritised research questions (arising from the gaps identified in the knowledge synthesis) were included in a report by the Public Health Institute.

#### Research capacity assessment

A review of the literature on capacity assessment was conducted. Two documents were developed from this review. The first focused on the different research system capacity assessment models available (18 models were found) and this review was included in a report on *Health Research Capacity in Sudan: A Need for Situational Analysis and Different Ways to Do It*. One of these models was used to assess the health research system in Sudan as part of a broader study in 2003 by the WHO Eastern Mediterranean Regional Office [30]. Given the resource constraints rather than conducting a capacity assessment survey a literature review was conducted based on published Sudanese health research. From this review the capacity to conduct health research in Sudan was assessed and the results of the review was compiled in the second report on *Health Research Capacity in Sudan: A Review of Available Literature.*

This research capacity assessment was conducted as a MSc in Public and Tropical Health Programme project at UMST. Interested MSc students were interviewed and one student from a group of 10 was selected as the appropriate candidate to complete the work under the supervision of UMST staff. This step gave ChRAIC Sudan an extra dimension of capacity building of postgraduate students at UMST and proved to be an excellent way to complete the work without the need to wait for the whole team of Sudan to be actively present, since all team members held very senior positions either in the FMoH or their respective academic institutions and finding a suitable meeting time would have been difficult.

#### Ph.D research

The Ph.D student used novel participatory research methods (Participatory Ethnographic Evaluation and Research - PEER) in conducting qualitative research for his doctoral thesis on access to maternal healthcare in post-conflict South Sudan. The student completed and graduated with his Ph.D in 2015.

**Fig. 3:**
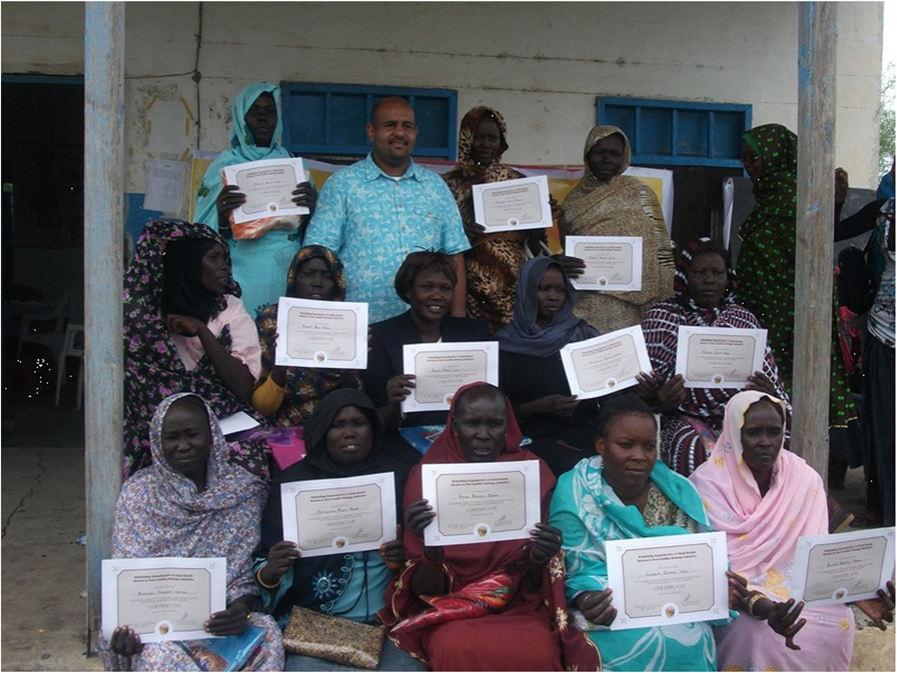
Ph.D researcher with PEER trainees, South Sudan

The Ph.D research took place in South Sudan in a county bordering Sudan. Fourteen illiterate women from 14 villages in Renk county in South Sudan were trained as PEER researchers (Fig. 3). The 14 trainees and the Ph.D student together developed the research questions and made drawings to help the trainee interviewers remember the questions. The trainee interviewers returned to their villages, and each of them interviewed three of their friends in their social networks on three different themes. Debriefing sessions were held on three occasions over 6 weeks. A final analysis workshop to help analyse the data was held where drama and stories were used as the main means of communication. The Ph.D student additionally conducted several focus group discussions, in- depth interviews and critical incidence analysis of maternal deaths, maternal near misses, neonatal deaths, and abortions.

**Fig. 4:**
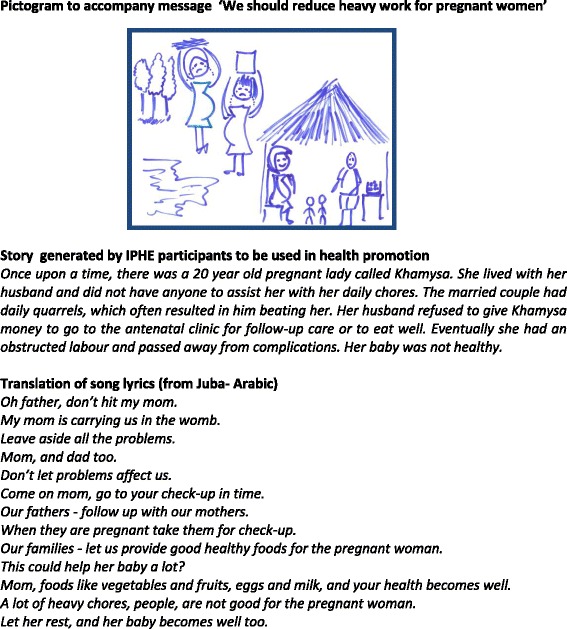
Examples of material developed to be used by PEER researchers and theatre groups to engage with communities

Based on the experience and contextual understanding gained from this data, two participatory action research interventions were identified to effectively engage two groups (community members and senior government officers) in the promotion of women’s health in Renk County in South Sudan. The first intervention was a form of ‘innovative participatory health education’ (IPHE) [18, 31]. Ten of the 14 PEER researchers worked together with two employees of a local NGO and 10 local theatrical band members to identify important issues related to women’s health in their community. They developed context-friendly material, which they presented to their community in the form of pictograms, songs and drama (see Fig. 4).

The second intervention was in the form of ‘participatory reproductive health project management’. Ten senior officers in the Health Department of Renk county were chosen to strengthen their capacity to develop, implement, monitor and evaluate reproductive health projects. They used the list of maternal health issues generated by the PEER researchers to develop two reproductive health project proposals. In the last day of the workshop, the IPHE participants and the senior government officers came together to discuss maternal health issues in the area. The senior officers presented the two proposals they had worked on to the IPHE participants, who in turn gave them feedback and comments. At the end of the training the senior officers had further developed the two proposals based on the issues raised in the research and also on the feedback of the community members. The proposals focused on youth awareness on reproductive health, and; the rehabilitation of the reproductive health centres in Renk county.

### Impact at local, national and global levels

#### Local impact

The Ph.D research has had an impact on the PEER researchers and health promoters, the health service officials and the community in which they work. Some of the examples of change which were highlighted by the PEER participants are:

- *Changes in perception of pregnancy*: IPHE participants said they saw pregnancy with new eyes. Pregnancy and labour were no longer viewed with indifference, but were now considered significant events. They noted that they have a better understanding of situations in which pregnant women require care.
- *Change is possible*: IPHE participants used to feel overwhelmed by maternal health concerns. While women get pregnant every day, and many go through uneventful pregnancies, others develop complications and may die. They felt helpless and unable to do anything to change the situation. They now felt that they can make gradual progress by addressing the issues that can feasibly be changed and that they could have a measurable impact on maternal health.
- *Empowerment:* IPHE participants stated that this experience has influenced their personal behaviour. Actively participating throughout the different phases of this intervention and the knowledge they have gained from this experience has resulted in them being empowered and motivated to take action around their own health and seeking health care.

Senior staff from the Renk County Department of Health, South Sudan, who participated in the proposal development workshop recognised that such a participatory approach strengthened their relationship with the local people. They also felt that the process enhanced the contribution of marginalised communities in identifying needs, and in planning and designing future health services in this post-conflict setting. They also noted that this approach helped them to identify the maternal health issues through the lens of the local community and that this will influence their future decision-making process. Additionally, they expressed how the process had enhanced their own confidence and competency through acquiring some of the basic skills to manage a project.

Community members interviewed felt that their awareness of maternal health issues increased through the sharing of information in a simple and attractive way: the song and the drama were performed in the local language, and the material was generated by members of  the local community coming from the same culture. The community found this approach mirrored their lives and it was easier to connect with than other health promotion materials or events they had previously encountered or attended. They didn’t feel threatened or reprimanded and felt that the messages were displayed and communicated in a culturally-appropriate manner. They also stated that they would now be more willing to change behaviour as they recognised how behaviour can adversely influence outcomes.

#### National impact

**Fig. 5:**
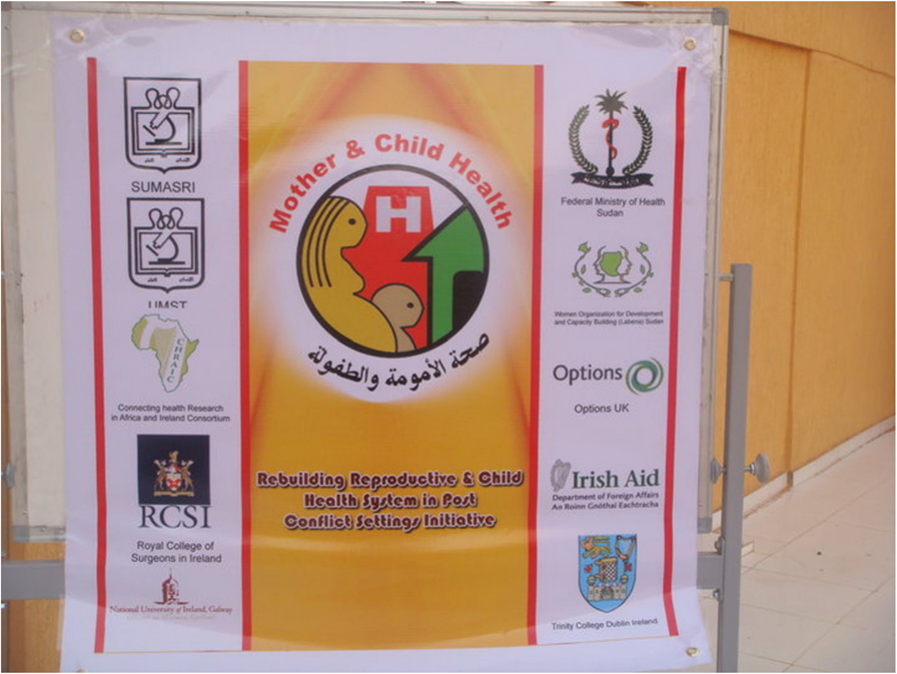
Banner for RCRU used at dissemination events
- *Research capacity strengthening*
  Building on the experience of the knowledge synthesis and policy dialogue, UMST introduced qualitative methods as a component of the curriculum of the existing postgraduate diploma in research methodology and biostatistics in 2011. The qualitative research module has been delivered to 75 Masters and Ph.D students at UMST and 20 students conducted field work for 2 weeks in South Sudan. Eight UMST students were also trained on PEER with the help of *Options UK*. The Ph.D student attributes his ability to do this work to the knowledge, skills, and competencies that he developed during the taught component of the Ph.D programme. As the final evaluation of CHRAIC report notes concerning this and other ChRAIC PhD students :
  *In building the capacity of these individuals ChRAIC has facilitated an informal cascade of capacity strengthening, as skills and knowledge learnt through the project should be passed on to subsequent generations of students (Independent end of project evaluation report)*
  WHO, Sudan, requested that the UMST teach qualitative methods as a standalone course for staff and other members of the public who would wish to develop  qualitative research skills. The importance of this move is highlighted in the quote below from the mid-term evaluation.
  *This is the first time to launch a qualitative research module in the postgraduate diploma in research methodology. So this is for the first time and we teach more innovative ways to do qualitative research, like PEER. So this is one of the big achievements that we have because people here they are not used to qualitative research and believe qualitative research is either in-depth interviews or focus group discussions. So we found many difficulties in convincing people that we need to teach such new approach and now it is actually very good. (ChRAIC Sudan member, Mid-term Evaluation)*

- *Policy Dialogue*
  There are clear and directly attributable impacts of the ChRAIC research process and outputs on national policy dialogues and policies in Sudan. Firstly, the North Sudan ChRAIC research process and team composition were shared as examples of researchers/policy makers partnership in a Policy Brief Writing Workshop, conducted by the Health Policy Directorate of the FMoH, the WHO Country Office and participants from McMaster University-American University of Beirut in October 2011. A policy brief titled “Promoting Access to High Quality Primary Health Care Services in Sudan”, was the direct result of this workshop.
  Secondly, ChRAIC Sudan was involved in the preparation of the National Health Research Policy Brief 2013, The National Nutrition Policy Brief 2013 and were consulted on the National Health Insurance Policy. The National Health Research Policy and Sudan Country report 2012 explicitly refers to ChRAIC priority setting exercise.
  Thirdly, the systematic approach to conducting literature reviews that was used in the knowledge synthesis exercise has been used to generate data for other projects in the FMoH indicating capacity developed for more evidenced based approaches to intervention design and policy development.
- *Institutional linkages made nationally*
  As noted above UMST had two Memorandum of Understanding: one with the FMoH and the other with EPI LAB in Khartoum, Sudan. The Memorandum of Understanding with EPI LAB served the purpose of getting data on HIV/AIDS and TB for the Sudan knowledge synthesis report, but additionally facilitated training of postgraduate students. The  strong link with EPI LAB has opened doors for postgraduate students from UMST to carry out their research projects. Commenting on the EPI LAB Memorandum of Understanding a member of the steering committee in the mid-term evaluation noted:
  *I think this (the Memorandum of Understanding) has opened for us new avenues. Our students are going now to the Ministry of Health and our other students are going to the EPI LAB (the epidemiology lab) to do their degrees or collect data from these institutions, and vice versa. The ministry is approaching us to do some work and also the epidemiology lab is doing the same. So we are now opening to other institutions and other interested parties to come in because of this activity. (ChRAIC North Sudan member, Mid-term Evaluation)*
  In 2010, UMST in collaboration with ChRAIC established the Reproductive and Child Health Research Unit (RCRU) as a ‘think tank’ and leading centre for conducting research on maternal and child health in Sudan. Membership of RCRU currently stands at 2100. The aim of RCRU is to produce evidence and to do research with hard-to-reach communities in conflict affected fragile states, and to support evidence based health system decisions and policies. The RCRU website was developed in 2012 (http://www.rcru.org) and was designed as a means to establish a user friendly platform that was open to all and where relevant publications, documentation and activities on reproductive and child health could be shared publically (Fig. 5). The RCRU Facebook page has proven to be very successful with more than 1700 followers and it is this Facebook group that is now becoming the platform for discussing different health-related issues worldwide.

#### Global impact

The IPHE approach of ChRAIC Sudan was recognised worldwide by Women Deliver, a global advocacy organisation, on March 2012 as one of the top ten Educational Initiative Ideas and Solutions for improving the lives of girls and women. IPHE has also been identified by Tropical Diseases special programme as an example of a qualitative implementation research approach for improving the lives of girls and women worldwide [32].

RCRU, as the representative of the Geneva Foundation for Medical Education and Research in Sudan and a member of WHO ‘The Partnership for Maternal, Newborn, and Child Health’ and the Global Health Workforce Alliance, is an example of one collaborative linkage between Sudan and the Global Health arena that emerged from this project. Another example is Edulink. Edulink is an EU financed project where several African Institutes of research have developed a cross border network to investigate African migration and gender in the global context. ChRAIC Sudan team members are now part of this project.

Through the links established in ChRAIC, in June 2012, a number of senior stakeholders met at the Royal College of Surgeons in Ireland (RCSI) for a meeting organised by the Sudanese Medical Association (UK and Ireland) and the Health Service Executive of Ireland, with support from ChRAIC, the Irish Forum for Global Health and RCSI. The meeting was attended by representatives from: the Sudan Medical Council, the Sudan Medical Specialisation Board, the National Human Resources for Health Observatory, Sudan, the Sudan Medical Association, Sudanese Ph.D students, RCSI, Irish Aid, the Health Service Executive of Ireland, the College of Surgeons in East, Central and Southern Africa, the Irish Forum for Global Health and ChRAIC. The focus of the meeting was on medical training to address human resources for Health, with the overall aim of the meeting to discuss opportunities for collaboration around Post Graduate training of medical doctors and other forms of training/capacity building. The ultimate objective of the meeting was to develop a structured and effective collaboration between Sudan and Ireland on medical capacity building.

## Discussion

As outlined above achievements of the ChRAIC project in Sudan included a national knowledge synthesis on equity and access, governance and human resources in health; identification of country level health systems research priorities; research capacity assessment and skills training, and; the training and graduation of a Sudanese team member with a Ph.D. Mechanisms established in Sudan to facilitate these achievements included a Memorandum of Understanding at country level between the Ministry of Health, research and academic institutions in Sudan, and the establishment of country level initiatives and a research unit. The latter recognised globally through awards and membership in global health forums. Most of these achievements were due to ChRAIC Sudan being part of a multi-country partnership.

Regarding public advocacy value, it is hard to see how these achievements could have been attained without all the partners involved. The role of different partners  is evident above in the previous discussion of national policy but is also the case for the research conducted in Renk with the IPHE participants and the local government officials. The IPHE participants were able to communicate the main findings and messages through theatre and dance as well as being involved in a forum for influencing local interventions and strategies. The local government officials also appreciated the creation of an environment for community engagement and their input on the proposed projects. At a global level the recognition of the IPHE approach as one of the top ten Educational Initiative Ideas and Solutions for improving the lives of girls and women by Women Deliver illustrates how local and global can be connected through a networked approach.

ChRAIC Sudan certainly strengthened research capacity at many levels, through the PEER training of the local women and master students as well as the more long term effect of including qualitative research into an existing postgraduate diploma. The documentation of the processes for conducting the knowledge synthesis and the research prioritisation have also enabled the learning to extend beyond the ChRAIC team. As this paper illustrates the process and outcomes can be considered as a means of capacity strengthening for researchers and policy makers, and as an approach for knowledge translation and multilevel capacity building, and addressing real problems regarding women’s’ health. The levels of capacity development is illustrated in Table 1 (adapted from [26]) .

**Table 1:** Matrix of capacity building strategies in ChRAIC Sudan

	ChRAIC approach to capacity building			
Entity targeted	Graduate or post graduate training	Learning by doing	Institutional partnerships between higher and lower income countries	Centres of excellence
Individual & Community (Local)	✓ **PhD scholarships** ✓ Masters students receive PEER and qualitative research training	✓ **Action research using PEER by PhD student** ✓ **PEER training** and research conducted by 14 community women	✓ ChRAIC PhD student agreement	
Institution	✓ Qualitative research and PEER training in Masters Curriculum at UMST	✓ Renk county senior department of health officials involved in proposal development ✓ Local theatre groups involved in drama for health promotion	✓ ChRAIC cross country institutional partnership	
National			✓ Memorandums of Understanding signed: – UMST and EPILAB – UMST and FMoH	
Global		✓ RCRU as a ‘think tank’ and leading centre for conducting research on maternal and child health in Sudan established.		✓ IPHE recognised by Women Deliver in 2012 as one of the top ten Educational Initiative Ideas and Solutions for improving the lives of girls and women. ✓ IPHE has also been identified by Training in Tropical Diseases special programme and the WHO as an example of a qualitative implementation research approach for improving the lives of girls and women worldwide

Bold indicates where financial support came from the research project

Additionally, many of the constraints faced by the ChRAIC team could not have been addressed without the pooled human, institutional and financial resources. With this support delays in funding were mitigated, access to documents and data facilitated, and sharing of lessons learned beyond those who were members of ChRAIC.

Revisiting the ‘network of action’ approach adopted in this partnership we believe that this approach facilitated a more enabling environment in which these benefits could be realised. The first element of ‘abandoning singular, one-site’ projects ([21], p359) in establishing ‘networks of action’ is evidenced in the numerous partners in ChRAIC – Lesotho, Malawi, Mozambique, Sierra Leone, South Sudan, Sudan, Uganda. However, this principle was also applied at country level. Each country had to establish country research teams and these were to be formed through a network of academics, research institutions and, developmental and governmental organisations - evidenced above in the composition of the Sudan ChRAIC team.

From the start, there was agreement in terms of the broad focus of the ChRAIC project, and what was to be delivered, namely the focus was on health systems research capacity strengthening in the areas of governance, equity and access, and human resources. However, there was a great deal of flexibility and resultant variety regarding the process, such as the local administration and governance of the ChRAIC country research teams, as well as how the knowledge synthesis was to be conducted. Additionally, as the research prioritisation and strengthening was context specific the exact health systems research conducted was based on the results of local research priorities and capacity gaps. This is in line with the second element of a ‘network of action approach’ ‘generating local, self-sufficient learning processes’([21], p359). As noted above the detailed process of conducting the knowledge synthesis and the process of establishing the partnership have been disseminated and utilised by others in Sudan. Practical demonstrations on how to search for data, which databases to use and how they can be accessed, and on how to conduct literature reviews were given in the first annual global ChRAIC workshop. In the second annual global ChRAIC workshop country presentations on completed knowledge synthesis reports, research prioritisation and capacity analysis techniques were given. In the interim period guidelines on conducting a knowledge synthesis were developed by the global ChRAIC coordinator and included illustrated examples of the process from the countries that had completed that stage of activities. Unfortunately, as noted in the independent end of project evaluation, several stakeholders reported they had felt a crucial lack of guidance during the initial phase of the project, and sensed that partners ‘weren’t on the same page' concerning  the expectations related to the knowledge synthesis and its report. Flexibility and variety are not without challenges.

One of the principles in establishing a network of action is to nurture a ‘robust, heterogeneous collection of actors likely to pursue distinct, yet sufficiently ‘similar’… agendas’([21], p359). This partnership illustrates how the slow process of evolving the country team and commitment to get people on board and involved from an early stage enabled the development of long term relationships and agreements that have continued beyond the life of ChRAIC. As one of the steering group members noted:

*I think we did good work in marketing the idea, engaging policy makers from the early stage so that they feel the ownership, they feel the responsibility and they feel that this is their own project. It’s not like other projects where we are recruiting them to do a project, but from the start we built this ownership and responsibility and we marketed the idea in a good way to engage them at the early stage. And sometimes, you know, we accepted delay because we feel that this will help engaging more policy makers and it spreads the idea about this collaboration. (ChRAIC North Sudan member, Mid-term Evaluation)*

Without the final element of ‘aligning interventions with the surrounding configurations of existing institutions, competing projects and effort as well as everyday practices’ ([21], p359) many of the challenges faced over the course of the project could not have been overcome. For example, most of the literature needed for the knowledge synthesis was with the FMOH. However, as UMST is a private university it was difficult to get governmental resources to support the process, as and when needed. Through alignment of needs and illustration of benefits within a Memorandum of Understanding this challenge was overcome. Likewise, due to the difficulties of transferring money electronically to Sudan the UMST hosted the meetings at its own expense and the team members received no payment for their time or for costs incurred in the process of producing the knowledge synthesis. Meetings were also planned for Saturdays to minimise disruption to everyday work, but also indicate the serious commitment of the people involved.

## Conclusion

It was recognised in the independent evaluation of ChRAIC that many of the recognised principles of partnerships, such as trust, respect, and regular communication were adhered to across the project. However, this does not explain why the linkages between the different elements of the project and the impact of the project at the national level seem to have had a greater multiplier effect in Sudan than the other ChRAIC country projects. The same principles of establishing a network of action were applied to all countries. The success could partly be explained by the perhaps opportunistic strategic linkage that was able to be made between the Ph.D programme and the in-country ChRAIC team.

*‘That person is a Sudanese involved in ChRAIC programme doing his Ph.D with ChRAIC - and for us they build the capacity of that Ph.D student, but he came back…two or three times a year when he would teach in the department of research methodology, specifically qualitative research.’ (Independent end of project evaluation interviewee)*

However, this still does not explain why other Ph.D students located in the same institution or in nearby institutions to the lead ChRAIC country partner did not make the same connections. Many of the reasons for the strong connection are attributable to the individual Ph.D student and the commitment of the lead partner in Sudan. In other countries where the student was also based in the lead institution these close ties did not occur (4 out of the 8 Ph.D students were in this situation). Another possible explanation for the differences is that the process in Sudan went much slower than in some other countries. The slow progress  meant that there was time for the Ph.D students’ research to evolve alongside the establishment of the country ChRAIC team. Additionally, it may also be that while the local health systems capacity can be considered to have been lower in Sudan, there was also a much stronger perceived need locally for these interventions. Other partner countries  were in more well research resourced and donor -crowded terrains with stronger health systems research capacity and therefore may not have prioritised this resource. This position is linked to the fact that some of the partners within the ChRAIC programme were institutions that have multiple international research partners with multiple concomitant demands on limited resources. The implication being that though these partners can be viewed as being better resourced regarding the level of skills and finances, the number of commitments and demands on these individuals and institutions can cause serious constraints in the capacity to deliver on all their commitments.

Despite the same ‘network of action’ approach to developing the partnership across numerous African countries the Sudan ChRAIC team not alone delivered the planned outputs, but also had more sustainable network and capacity effects. So though we agree there are benefits to partnerships as noted in the literature and the ‘network of action’ approach has its benefits, partnering in this manner may be necessary to obtain the benefits, but is not sufficient. The context and individuals involved led to different strategies and actions that enabled more sustainable networks and processes. These strategies and actions included: the strategic linkage made between the Ph.D programme and the in-country ChRAIC team; the high level of commitment of the country partners (at an institutional and individual level) to the process, and; the buy-in that was achieved through the slow process of evolving the country team.

## Abbreviations

ChRAIC: Connecting health Research in Africa and Ireland Consortium
EPI LAB: Epidemiology Laboratory, Khartoum
FMOH: Federal Ministry of Health
HEI: Higher Education Institution
IPHE: Innovative Participatory Health Education
MDGs: Millennium Development Goals
NGO: Non-Governmental Organisation
PEER: Participatory Ethnographic Evaluation and Research
RCRU: Reproductive and Child Health Research Unit
RCSI: Royal College of Surgeons in Ireland
UMST: University of Medical Sciences and Technology
WHO: World Health Organisation

## Footnotes

1 ‘An chraic’ is the Irish term for ‘the fun/enjoyment’ and is also now used commonly in the English language.

2 Due to the instability in the country is was not until mid 2010 that a bank that would operate US dollar transfers to North Sudan was found by the RCSI finance department.
